# Angiotensin II stimulates superoxide production by nitric oxide synthase in thick ascending limbs

**DOI:** 10.14814/phy2.12697

**Published:** 2016-02-16

**Authors:** Agustin Gonzalez‐Vicente, Jagannath H. Saikumar, Katherine J. Massey, Nancy J. Hong, Fernando P. Dominici, Oscar A. Carretero, Jeffrey L. Garvin

**Affiliations:** ^1^ Department of Physiology and BiophysicsSchool of MedicineCase Western Reserve UniversityClevelandOhio; ^2^ Facultad de Farmacia y BioquímicaUniversidad de Buenos AiresCiudad Autónoma de Buenos AiresArgentina; ^3^ Department of Internal MedicineHypertension and Vascular Research DivisionHenry Ford HospitalDetroitMI; ^4^ Instituto de Química y Fisicoquímica BiológicasCONICETCiudad Autónoma de Buenos AiresArgentina; ^5^ Present address: Wayne State UniversityDetroitMichigan; ^6^ Present address: Division of NephrologyThe University of Tennessee Health Science CenterMemphisTennessee

**Keywords:** Kidney, NADPH oxidase, protein kinase C, reactive oxygen species, uncoupling

## Abstract

Angiotensin II (Ang II) causes nitric oxide synthase (NOS) to become a source of superoxide (O_2_^−^) via a protein kinase C (PKC)‐dependent process in endothelial cells. Ang II stimulates both NO and O_2_^−^ production in thick ascending limbs. We hypothesized that Ang II causes O_2_^−^ production by NOS in thick ascending limbs via a PKC‐dependent mechanism. NO production was measured in isolated rat thick ascending limbs using DAF‐FM, whereas O_2_^−^ was measured in thick ascending limb suspensions using the lucigenin assay. Consistent stimulation of NO was observed with 1 nmol/L Ang II (*P *<* *0.001; *n* = 9). This concentration of Ang II‐stimulated O_2_^−^ production by 50% (1.77 ± 0.26 vs. 2.62 ± 0.36 relative lights units (RLU)/s/*μ*g protein; *P *<* *0.04; *n* = 5). In the presence of the NOS inhibitor L‐NAME, Ang II‐stimulated O_2_^−^ decreased from 2.02 ± 0.29 to 1.10 ± 0.11 RLU/s/*μ*g protein (*P *<* *0.01; *n* = 8). L‐arginine alone did not change Ang II‐stimulated O_2_^−^ (2.34 ± 0.22 vs. 2.29 ± 0.29 RLU/s/*μ*g protein; *n* = 5). In the presence of Ang II plus the PKC *α*/*β*_1_ inhibitor Gö 6976, L‐NAME had no effect on O_2_^−^ production (0.78 ± 0.23 vs. 0.62 ± 0.11 RLU/s/*μ*g protein; *n* = 7). In the presence of Ang II plus apocynin, a NADPH oxidase inhibitor, L‐NAME did not change O_2_^−^ (0.59 ± 0.04 vs. 0.61 ± ×0.08 RLU/s/*μ*g protein; *n* = 5). We conclude that: (1) Ang II causes NOS to produce O_2_^−^ in thick ascending limbs via a PKC‐ and NADPH oxidase‐dependent process; and (2) the effect of Ang II is not due to limited substrate.

## Introduction

The reactive oxygen species nitric oxide (NO) and superoxide (O_2_^−^) have opposing physiological effects in the kidney. In the thick ascending limb of the loop of Henle, a nephron segment that is important in salt, water and acid/base homeostasis (Burg 1982; Greger 1985; Mount 2014), NO and O_2_^−^ regulate ion transport (Garvin and Ortiz 2003). NO promotes natriuresis and diuresis (Majid et al. 1993; Eitle et al. 1998; Plato et al. 1999; Ortiz et al. 2004b), whereas O_2_^−^ enhances Na reabsorption (Zou et al. 2001; Majid and Nishiyama 2002; Juncos and Garvin 2005; Juncos et al. 2006; Silva et al. 2006) and water retention. Imbalances between these two factors in the kidney can lead to renal pathophysiological conditions such as hypertension (Sedeek et al. 2003; Kopkan and Majid 2005; Kopkan et al. 2007; Majid and Kopkan 2007; Ramseyer et al. 2015) and progressive renal injury.

The thick ascending limb is one of the predominant sources of O_2_^−^ in the kidney (Zou et al. 2001; Li et al. 2002). In this segment, most of the O_2_^−^ is generated by NADPH oxidase 4 (Hong and Garvin 2012; Massey et al. 2012), a process involving several steps. Ultimately, an electron from NADPH is transferred to the oxidase subdomain and then to molecular O_2_, reducing it to O_2_^−^. Thick ascending limb NADPH oxidase activity is enhanced by a number of stimuli including luminal flow (Hong and Garvin 2012), ion delivery (Hong and Garvin 2007), and angiotensin II (Ang II) (Li et al. 2002). We have previously shown that Ang II stimulates NADPH oxidase‐dependent O_2_^−^ production by binding to Ang II type 1 (AT1) receptors and activating protein kinase C (PKC) (Herrera et al. 2010). We have also shown that once O_2_^−^ is generated, it can stimulate PKC activity and exert physiological effects including increasing Na reabsorption by thick ascending limbs (Silva et al. 2006). Although NADPH oxidase is the primary source of O_2_^−^ after Ang II stimulation, short hairpin RNAs against and inhibitors of NADPH oxidase only reduce Ang II‐enhanced O_2_^−^ by about 70% suggesting that other sources are involved. Potential sources include NO synthase (NOS), cyclooxygenase, and the mitochondria (Zou et al. 2001; Li et al. 2002).

Thick ascending limbs express all three isoforms of NOS (Mount and Power 2006). NO synthesis involves several steps. Ultimately, electrons are transferred from NADPH via FAD and FMN to the oxidase domain of NOS, which contains a heme group. These electrons are then passed from the enzyme to the substrate, L‐arginine, causing its reduction to L‐citrulline and release of NO. If substrate or cofactors are lacking, under some circumstances NOS can produce O_2_^−^ rather than NO because the electrons are transferred to molecular O_2_ rather than L‐arginine (Andrew and Mayer 1999). The balance between NO and O_2_^−^ production by NOS can be regulated by phosphorylation by a number of kinases including PKC (Forstermann et al. 1994; Fleming 2010; Herrera and Garvin 2010; Forstermann and Sessa 2012; Ramseyer et al. 2015). The NOS inhibitor L‐NAME blocks both NO and O_2_^−^ generation by NOS. Several stimuli can enhance NOS activity in thick ascending limbs including Ang II (Cabral et al. 2010, 2012; Herrera and Garvin 2010; Peng et al. 2014).

Similar to thick ascending limbs, Ang II stimulates both O_2_^−^ production by NADPH oxidase and NO production by NOS in endothelial cells. Ang II also causes NOS to produce O_2_^−^ (Mollnau et al. 2002; Oak and Cai 2007; Lobysheva et al. 2011; Lee et al. 2013; Galougahi et al. 2014; Saura et al. 2014) in endothelial cells. Thus, we hypothesized that Ang II induces O_2_^−^ production by NOS in thick ascending limbs via a PKC‐ and NADPH oxidase‐dependent process.

## Materials and Methods

### Animals

All protocols in this study were approved by the Case Western Reserve University and the Henry Ford Hospital Institutional Animal Care and Use Committees. All experiments were conducted in accordance with the National Institutes of Health Guidelines for the Care and Use of Laboratory Animals. Male Sprague–Dawley rats (Charles River Breeding Laboratories, Kalamazoo, MI) were fed a diet containing 0.22% Na^+^ and 1.1% K^+^ for at least 6 days prior to the experiments. For terminal surgery, animals were anesthetized with ketamine (100 mg/kg bw IP) and xylazine (20 mg/kg bw IP), and given 2 IU heparin (IP). Animals were killed while still under anesthesia.

### Drugs and buffers

Unless specified, all drugs and reagents were obtained from Sigma‐Aldrich (St Louis, MO). The cell‐permeant NO‐selective fluorescent dye DAF‐FM‐diacetate was purchased from Invitrogen (Grand Island, NY). Coomassie Plus Protein Assay Reagent was obtained from Thermo‐Scientific (Rockford, IL).

HEPES‐buffered physiological saline contained (in mmol/L): 10 HEPES (4‐(2‐hydroxyethyl)‐1‐piperazineethanesulfonic acid) (pH 7.5 at 22°C), 130 NaCl, 4 KCl, 2.5 NaH_2_PO_4_, 1.2 MgSO_4_, 5.5 glucose, 6.0 DL‐alanine, 2.0 Ca(lactate)_2_, and 1.0 Na_3_citrate. Osmolality was adjusted to 300 ± 5 mOsmol/L with mannitol.

### Thick ascending limb isolation

Single medullary thick ascending limbs were isolated as previously described (Cabral et al. 2010, 2012). Briefly, animals weighing 120–150 g were anesthetized and the abdominal cavity opened. The left kidney was bathed in ice‐cold 150 mmol/L NaCl, and then, immediately removed and placed in HEPES‐buffered physiological saline at 4°C. Coronal slices were cut and individual thick ascending limbs isolated from the outer medulla under a stereomicroscope at 4–8°C. Tubules ranging from 0.5–1.0 mm were transferred to a temperature‐regulated chamber and maintained at 37 ± 1°C. The bath was exchanged at 0.6 ml/min.

### Thick ascending limb suspensions

Suspensions were prepared as described before (Gonzalez‐Vicente and Garvin 2013; Gonzalez‐Vicente et al. 2014). Briefly, kidneys were perfused retrograde *via* the abdominal aorta with cold HEPES‐buffered physiological saline containing 2 USP/ml heparin and 0.1% of Type 4 collagenase. Perfused kidneys were removed, coronal slices cut, and outer medullary tissue dissected and minced. Minced tissue was digested in 0.1% collagenase for 30 min at 37°C. During digestion, tissue was agitated and gassed with 100% O_2_ every 5 min. The sample was then centrifuged (100 × *g*, 2 min, 4°C), and the resulting pellet of tubules resuspended in fresh HEPES‐buffered physiological saline and stirred on ice for 30 min. After stirring, the sample was filtered through a 250 *μ*m nylon mesh, and the filtered tubules were collected and rinsed at 4°C. This preparation resulted in a 95% pure suspension of thick ascending limbs.

### NO production measurement

NO production was measured by fluorescence microscopy as previously described (Ramseyer et al. 2015). Briefly, manually dissected thick ascending limbs were transferred to a temperature‐controlled chamber (37 ± 1°C) on the stage of an inverted microscope and fixed in place using glass pipettes for live cell microscopy (Diaphot TMJ, Nikon, Japan). The bath contained 100 *μ*mol/l L‐arginine, but tubules were not submitted to luminal flow. Under these conditions, NO production is negligible (Ortiz et al. 2001, 2004a). Tubules were loaded with the NO‐sensitive fluorescent dye DAF‐FM (2 *μ*mol/L) for 15 min and then washed with physiological saline for 5 min. For imaging, a 40X oil immersion objective was used. The dye was excited by a xenon arc lamp with a 488 nm band pass filter. The fluorescence emitted by the NO‐bound dye (>500 nm) was measured using Metafluor software (Molecular Devices, Sunnyvale CA). Results were expressed as arbitrary fluorescent units (AFU)/min.

### Superoxide production measurement

O_2_^−^ production was measured with bis‐N‐methylacridinium nitrate (lucigenin). Lucigenin is a validated luminescent probe to measure O_2_^−^ (Skatchkov et al. 1999; Ohashi et al. 2015), and it has been used in our laboratory before (Ortiz and Garvin 2002; Herrera et al. 2010; Silva and Garvin 2010; Massey et al. 2012). Briefly, for each experiment, lucigenin (5 *μ*mol/L final concentration) was added to each of two plastic tubes containing 800 *μ*l of warm‐oxygenated physiological saline and kept in the dark at 37°C. Then, 200‐*μ*l aliquots of freshly prepared thick ascending limb suspension containing 100 to 200 *μ*g of protein were added to each tube. The control tube was placed in a luminometer (FB12/Sirius; Zylux Oak Ridge, TN) and basal luminescence recorded at 4.8 sec intervals for 10 min using the provided software. Then, the O_2_^−^ scavenger 4,5‐dihydroxy‐1,3‐benzenedisulfonic acid (Tiron) was added to a final concentration of 10 mmol/L, and measurements were continued for 5 min. The average luminescence of the last 2 min following the addition of Tiron was then subtracted from the steady‐state luminescence before Tiron. Tubules were recovered by centrifugation, and luminescence normalized for protein content.

In the experiments using Ang II, it was added before placing the tubes in the luminometer. In some experiments, we used the lipid‐dependent PKC activator phorbol 12‐myristate 13‐acetate (PMA) instead of Ang II, which was also added before placing the tubes in the luminometer. For each set of experiments, the order of the control and treatment tubes was randomized.

For the experiments involving either L‐arginine or L‐NAME, the chemical was added during the generation of the suspensions. When Gö 6976 or apocynin was used, it was added 3 min before the addition of Ang II.

### Statistical analysis

Results are expressed as the arithmetic mean ± the standard error of the mean. The Student t‐tests was used to compare means. The *P‐*values were calculated using two‐tailed tests in all cases, and paired or unpaired test were used where appropriate. *P < *0.05 was considered significant.

## Results

**Figure 1:**
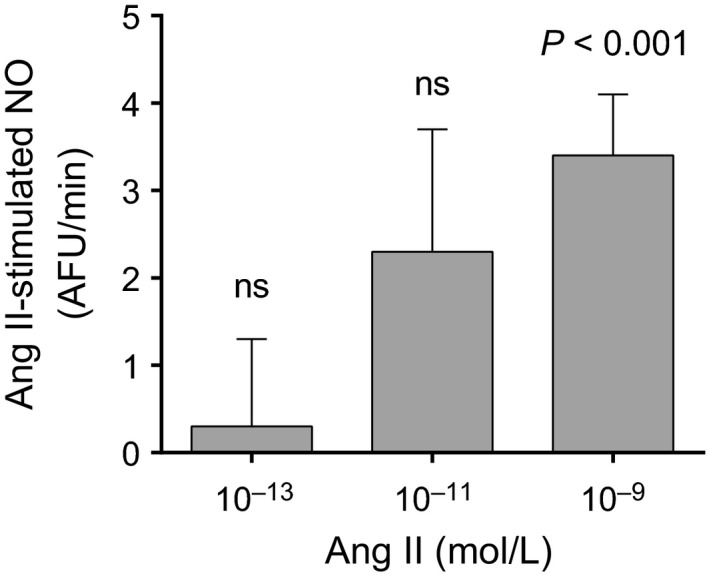
Effect of acute angiotensin II (Ang II) treatment on NO production by isolated thick ascending limbs. Tubules were manually dissected, held with pipettes and NO production measured with DAF‐FM. *P *< 0.001 versus basal; *n *= 9 for 1 nmol/L Ang II. AFU, arbitrary fluorescence units.

To show that Ang II activates thick ascending limb NOS and stimulates NO production, and to study which concentration of Ang II should be used in subsequent experiments, we measured the effect of different concentrations (10^−13^, 10^−11^, 10^−9^ mol/L) of Ang II on NO production by isolated thick ascending limbs. We found that within 5 min, 10^−9^ mol/L Ang II consistently increased NO production (*P *<* *0.001; *n* = 9), while lower concentrations gave a highly variable response (Fig. 1).

**Figure 2:**
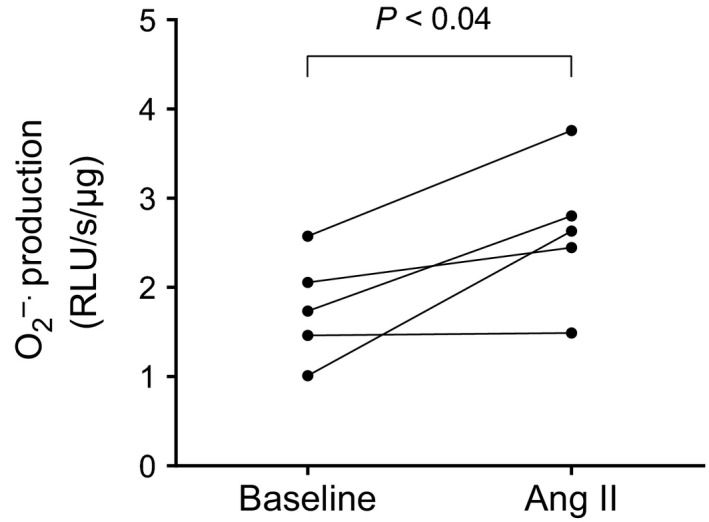
Effect of 1 nmol/L Ang II on O_2_^−^ production by thick ascending limb suspensions (*P *< 0.04; *n *= 5). Lucigenin plus Tiron were used to detect O_2_^−^ production in the presence or absence of Ang II.

Next, we tested whether a 5‐min treatment with 10^−9^ mol/L Ang II increased O_2_^−^ production in thick ascending limb suspensions in the absence of general NOS substrate L‐arginine, to avoid any potential interference from endogenously produced NO. We found that within 5 minutes, 10^−9^ mol/L Ang II increased O_2_^−^ production by about 50% from 1.77 ± 0.26 to 2.62 ± 0.36 relative light units (RLU)/s/*μ*g protein (*P *<* *0.04; *n* = 5; Fig. 2). Given that 5‐min treatments with 10^−9^ mol/L Ang II are capable of stimulating both NO and O_2_^−^, we used this timeframe and concentration in all other experiments.

**Figure 3:**
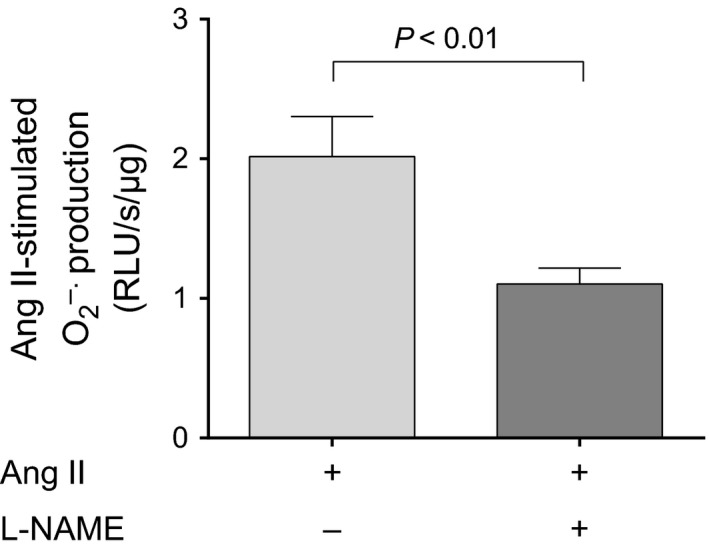
Effect of the NOS inhibitor L‐NAME (1 mmol/L) on Ang II‐induced O_2_^−^ in thick ascending limb suspensions (*P *< 0.01; *n *= 8). Lucigenin plus Tiron were used to detect Ang II‐stimulated O_2_^−^.

Ang II stimulates O_2_^−^ production by NOS in endothelial cells (Lobysheva et al. 2011). Thus, we next evaluated the contribution of NOS‐derived O_2_^−^ by measuring Ang II‐induced O_2_^−^ in the presence and absence of the general NOS inhibitor L‐NAME (1 mmol/L). These experiments were conducted in the absence of L‐arginine to avoid interference by NO. We found that Ang II‐induced O_2_^−^ production in tubules treated with vehicle was 2.02 ± 0.29 RLU/s/*μ*g protein, while in those treated with L‐NAME, it was 1.10 ± 0.11 RLU/s/*μ*g (*P *<* *0.01; *n* = 8; Fig. 3). Thus, blocking NOS reduces Ang II‐stimulated O_2_^−^ more than 40%.

**Figure 4:**
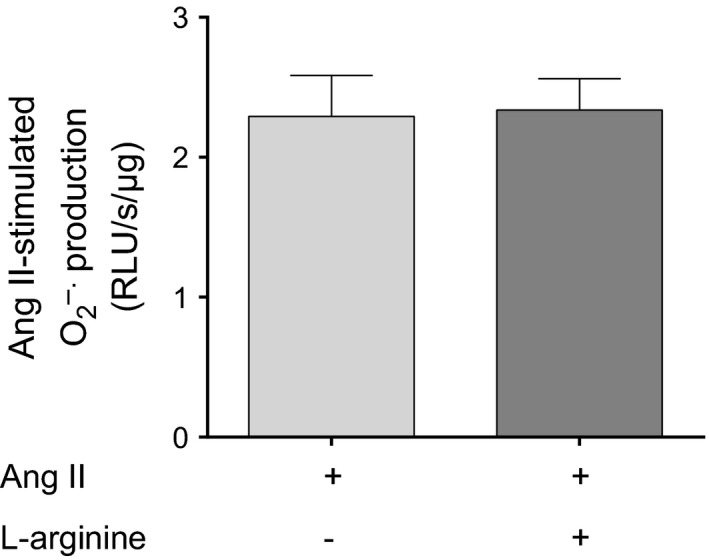
Effect of the NOS substrate L‐arginine (100 *μ*mol/L) on Ang II‐induced O_2_^−^ in thick ascending limb suspensions (*n *= 5). Lucigenin plus Tiron were used to detect Ang II‐stimulated O_2_^−^.

The results presented in Figure 3 indicate that Ang II stimulates O_2_^−^ production by NOS. This can be due to a direct effect or due to lack of substrate, that is, L‐arginine. To test whether Ang II‐stimulated NOS‐dependent O_2_^−^ production was a result of lack of L‐arginine, we repeated the experiments in the presence and absence of 100 *μ*mol/L L‐arginine. We found that Ang II‐induced O_2_^−^ production was not different in the presence (2.34 ± 0.22 RLU/s/*μ*g protein) or absence (2.29 ± 0.29 RLU/s/*μ*g protein) of L‐arginine (*n* = 5; Fig. 4). These data indicate that Ang II‐stimulated O_2_^−^ production by NOS is not only a consequence of the lack of L‐arginine to accept electrons, and thus other effects may be involved.

**Figure 5:**
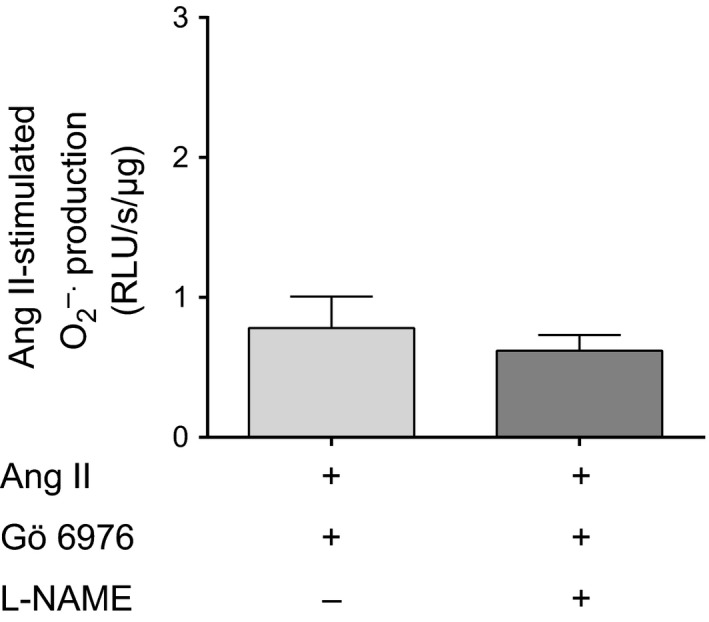
: Effect of the NOS inhibitor L‐NAME (1 mmol/L) on Ang II‐stimulated O_2_^−^ in thick ascending limb suspensions in the presence of the PKC *α*/*β*_1_ inhibitor Gö 6976 (100 nmol/L)(*n *= 7). Lucigenin plus Tiron were used to detect Ang II‐stimulated O_2_^−^.

Previous reports indicate that PKC activation causes NOS to produce O_2_^−^. Ang II can stimulate PKC activity in thick ascending limbs both directly (Herrera et al. 2010) and indirectly via O_2_^−^ (Silva et al. 2006; Herrera et al. 2010). Therefore, we next tested whether PKC activation was required for Ang II to increase NOS‐derived O_2_^−^, by measuring Ang II‐induced O_2_^−^ in the presence and absence of L‐NAME, while inhibiting PKC*α*/*β*_1_ with 100 nmol/L Gö 6976 (Fig. 5). We found that in the presence of Ang II and Gö 6976, O_2_^−^ production was unaltered by L‐NAME (0.78 ± 0.23 vs. 0.62 ± 0.11 RLU/s/*μ*g protein; *n* = 7). These data indicate that PKC activation is necessary for Ang II to stimulate O_2_^−^ production by NOS.

**Figure 6:**
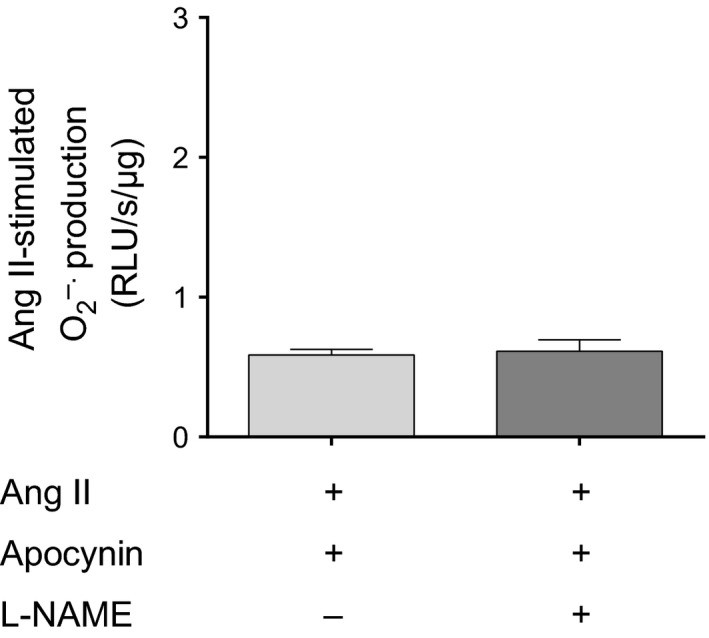
Effect of the NOS inhibitor L‐NAME (1 mmol/L) on Ang II‐stimulated O_2_^−^ in thick ascending limb suspensions in the presence of the NADPH oxidase inhibitor apocynin (10 *μ*mol/L) (*n *= 5). Lucigenin plus Tiron were used to detect Ang II‐stimulated O_2_^−^.

Ang II can also stimulate PKC indirectly by increasing O_2_^−^ derived from NADPH oxidase (Silva et al. 2006; Herrera et al. 2010). Apocynin reduces Ang II‐stimulated O_2_^−^ from NADPH oxidase (Herrera et al. 2010). Therefore, to test whether NADPH oxidase was involved in the ability of Ang II to stimulate O_2_^−^ by NOS, we measured Ang II‐induced O_2_^−^ in the presence and absence of L‐NAME, when NADPH oxidase was blocked with 10 *μ*mol/L apocyinin (Fig. 6). We found that in the presence of Ang II and apocynin, O_2_^−^ production was not affected by L‐NAME (0.59 ± 0.04 vs. 0.61 ± 0.08 RLU/s/*μ*g protein; *n* = 5). These data indicate that NADPH oxidase activation is a necessary event for Ang II to increase O_2_^−^ production by NOS in thick ascending limbs.

Finally, to test whether direct activation of lipid‐dependent PKC isoforms was able to stimulate O_2_^−^ production by NOS in our system, we measured the effect of PMA alone on O_2_^−^ production, and the effect of L‐NAME on PMA‐stimulated O_2_^−^ production in the presence of apocynin. We found that PMA‐stimulated O_2_^−^ production from 1.5 ± 0.3 to 12.8 ± 2.8 RLU/s/*μ*g protein (*P* < 0.05, *n* = 5). L‐NAME reduced PMA‐stimulated O_2_^−^ production in the presence of apocynin by 49 ± 15% (*P* < 0.05, *n* = 4). These data indicate that lipid‐dependent PKC isoform activation increases O_2_^−^ production by NOS in the absence of NADPH oxidase‐derived O_2_^−^ in thick ascending limbs.

## Discussion

We hypothesized that Ang II causes NOS to become a source of O_2_^−^ and that this effect is dependent on PKC and NADPH oxidase. This implies that Ang II stimulates both O_2_^−^ and NO production. Therefore, we began our study by directly measuring the effect of different concentrations of Ang II on NO production by rat thick ascending limbs. We found that 1 nmol/L Ang II stimulates thick ascending limb NO production within 5 minutes. Using this concentration of Ang II, we next tested whether it also stimulates O_2_^−^ production. We found that 1 nmol/L Ang II stimulated O_2_^−^ by approximately 50% in the same time frame as it stimulates NO. Taken together, these data suggest that there is a concomitant production of NO and O_2_^−^ upon Ang II stimulation in the thick ascending limbs. The O_2_^−^ experiments were performed in the absence of L‐arginine, the substrate for NOS, because NO and O_2_^−^ can react to form peroxynitrite if they are generated in the same compartment at the same time (Pryor and Squadrito 1995; Squadrito and Pryor 1995) and we have shown that NO can inhibit O_2_^−^ production via a cGMP‐dependent mechanism. The results showing that Ang II stimulates both NO and O_2_^−^ in the same time frame are consistent with our previous studies in which we found that Ang II stimulates NO (Herrera and Garvin 2010) and O_2_^−^ (Herrera et al. 2010; Massey et al. 2012). They are also consistent with findings of other investigators (Mori and Cowley 2003; Lobysheva et al. 2011).

Although it is generally accepted that Ang II stimulates O_2_^−^ in the thick ascending limb primarily by NADPH oxidase, this enzyme is not the only source of Ang II‐stimulated O_2_^−^ in this nephron segment. We and others have shown that other sources account for 10 (Mori and Cowley 2003; Herrera et al. 2010) to 40% (Massey et al. 2012) of the O_2_^−^ produced by thick ascending limbs upon Ang II stimulation. However, in the previous studies, the identity of such sources was not determined. Therefore, we next investigated the contribution of NOS to the increase in O_2_^−^ production caused by Ang II by studying whether L‐NAME, a NOS inhibitor, alters the ability of Ang II to enhance O_2_^−^ production. We found that blocking NOS reduced Ang II‐stimulated O_2_^−^ by 40%. These data suggest that Ang II stimulates NOS to produce O_2_^−^.

Our results showing that Ang II causes NOS to produce O_2_^−^ are similar to reports in other cell types (Lobysheva et al. 2011; Lee et al. 2013; Galougahi et al. 2014; Kossmann et al. 2014; Saura et al. 2014). NOS has been shown to be a source of O_2_^−^ after Ang II treatment in endothelial cells (Lobysheva et al. 2011). Further support of this concept comes from studies in Ang II‐induced hypertensive animals. For instance, NOS3 was upregulated at both the RNA and protein levels in the myocardium (Tambascia et al. 2001) and vessels (Mollnau et al. 2002; Sullivan et al. 2002) of Ang II‐treated rats as compared to controls. However, these animals presented a marked decrease in vascular NO bioavailability as well as endothelial dysfunction, which is associated with elevated O_2_^−^. Thus, elevated NOS does not necessarily lead to an increase in NO, and NOS‐derived O_2_^−^ may represent a mechanism whereby Ang II causes prolonged oxidative stress (Oak and Cai 2007).

An alternative explanation for our data could be that Ang II‐stimulated NO production is required to activate O_2_^−^ production from another source such as xanthine oxidase, cyclooxygenase, or the mitochondria. This seems unlikely because neither xanthine oxidase nor cyclooxygenase activities have been shown to be affected by NO or cGMP. While mitochondrial respiration has been shown to be regulated by NO, it reduces oxygen consumption by this organelle rather than increasing it (Boveris et al. 2003).

In our experiments, we showed that Ang II causes NOS to produce O_2_^−^ in the absence of exogenously added L‐arginine. Lack of substrate causes the active enzyme to transfer electrons not to L‐arginine but to molecular oxygen thereby generating O_2_^−^. Thus, we tested whether Ang II‐stimulated O_2_^−^ was altered in the presence or absence of L‐arginine. We found that Ang II‐induced O_2_^−^ production was not significantly altered by L‐arginine. These data indicate that a simple lack of NOS substrate is not the main mechanism mediating the increase in O_2_^−^ in our experimental conditions.

Since NO inhibits O_2_^−^ production and that NO and O_2_^−^ can react to form peroxynitrite in isolated perfused tubules, one might ask why adding L‐arginine to the suspension media did not alter Ang II‐stimulated O_2_^−^ production via one of these pathways. The explanation is that unlike the isolated, perfused tubule preparation, where the bath is >10^6^ times the volume of the tubule and L‐arginine is being washed out continuously, the thick ascending limb suspensions are always in a restricted space. This allows the tubules to retain enough endogenous L‐arginine to support NO production during the experiment, given that the K_½_ for L‐arginine is only about 2 *μ*mol/L, and at the time of isolation, the thick ascending limb cells may contain as much as 300 *μ*mol/L L‐arginine.

A similar consideration should be made for the cofactor tetrahydrobiopterin (BH_4_). Even though reductions in BH_4_ can cause O_2_^−^ production by NOS (Bec et al. 1998; Andrew and Mayer 1999; Adak et al. 2000), this seems unlikely in our experimental conditions. Addition of the BH_4_ precursor sepiapterin to thick ascending limb suspensions has no effect on NO production (Herrera et al. 2006). This indicates that BH_4_ is not a limiting factor in our preparation. Thus, although BH_4_ levels are affected in models of chronic hypertension (Landmesser et al. 2003), including those dependent on Ang II (Kang et al. 2011), there is no evidence that acute exposure to Ang II *per se* reduces BH_4_ levels. Thus, reduced cofactors availability is not likely the cause of NOS‐derived O_2_^−^ in this case.

Because Ang II activates PKC both directly and indirectly, and PKC activation can increase O_2_^−^ production from NOS in other cells (Chen et al. 2014), we next tested whether PKC was necessary for Ang II to increase O_2_^−^ production from NOS in thick ascending limbs. We found that when PKC was blocked, L‐NAME had no effect on Ang II‐stimulated O_2_^−^. The importance of PKC as a mediator of O_2_^−^ production is in agreement with our previous studies in thick ascending limbs (Silva et al. 2006; Herrera et al. 2010; Hong et al. 2010). However, this study is the first to identify a role in NOS‐derived O_2_^−^ production.

Ang II can indirectly activate PKC by stimulating NADPH oxidase activity. To test whether NADPH oxidase is required for Ang II to stimulate O_2_^−^ production by NOS, we used apocynin. We found that apocynin prevented Ang II from enhancing O_2_^−^ production by NOS. These data indicate that NADPH oxidase activity is required for Ang II's effect on NOS.

**Figure 7:**
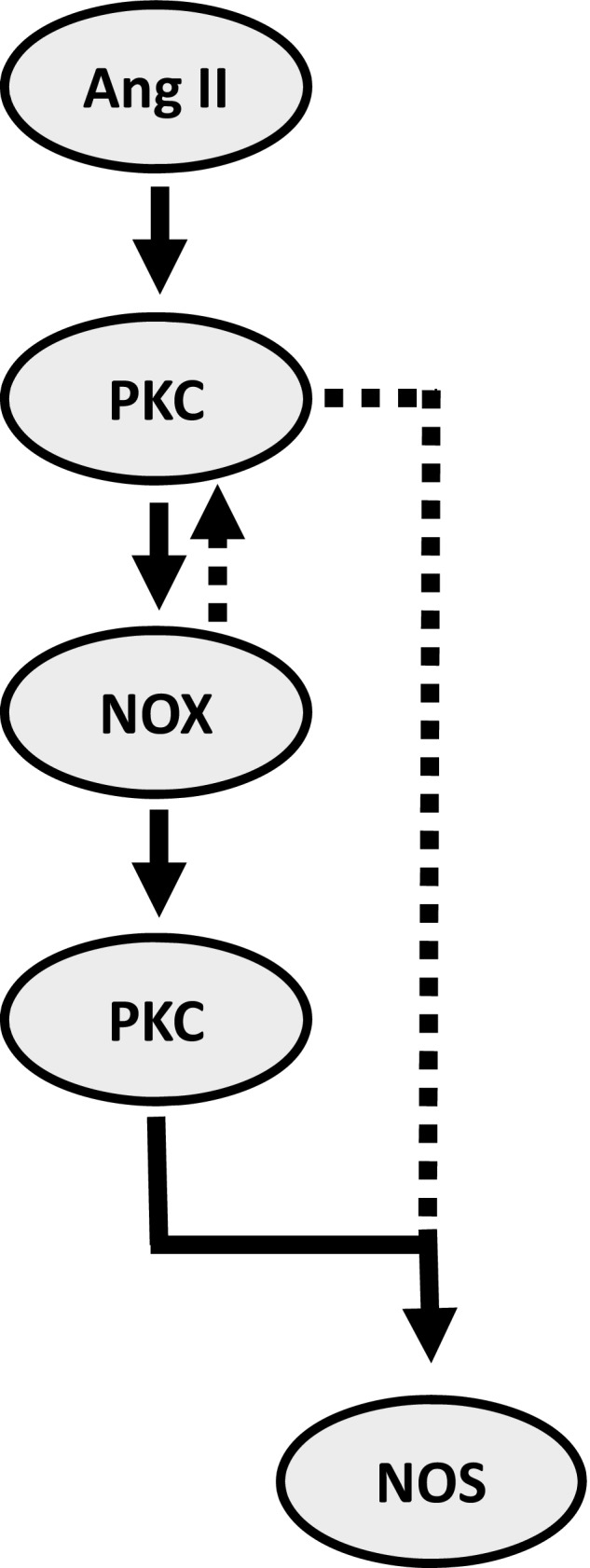
Ang II‐stimulated O_2_^−^ production by NOS requires at least one of these pathways involving NADPH oxidase: (1) NADPH oxidase‐derived O_2_^−^ exerts a positive feedback over the PKC *α* pool stimulated by Ang II (dashed lines); or (2) NADPH oxidase‐derived O_2_^−^ stimulating a different pool of PKC *α*/*δ* (solid lines). Both pathways converge in the final step which is NOS phosphorylation by PKC.

When taken together with published studies, the current PKC, apocynin, and PMA data suggest two possible pathways by which Ang II treatment can lead to O_2_^−^ production by NOS. Ang II binds AT1 receptors which activate PKC*α* (Herrera et al. 2010). PKC*α* then increases NADPH oxidase activity (Herrera et al. 2010; Hong et al. 2010; Massey et al. 2012). The O_2_^−^ thus produced either: (1) further activates the same pool of PKC*α* which increases NOS phosphorylation; or (2) activates a different pool of PKC*α*/*δ* (Silva et al. 2006). PKC*α*/*δ* then phosphorylates NOS causing it to produce O_2_^−^. Therefore, according to this model, the PKC*α* directly activated by Ang II does not cause NOS to produce O_2_^−^ because either: (1) it is in a different cellular compartment than the one that phosphorylates NOS; or (2) Ang II may simply not increase PKC*α* sufficiently to affect NOS. The proposed model is represented in Figure 7.

Another open question that cannot yet be answered is which NOS isoform is responsible from O_2_^−^ production in response to Ang II; however, some conclusions can be drawn based on published studies. First, NOS2 is mainly regulated at the transcriptional level and its abundance is at the limit of detection under nonstimulating conditions in the rat kidney (Zhang et al. 2000; Stumm et al. 2002), thereby making it unlikely to mediate any effect in acute experiments. Second, NOS1 is not phosphorylated by PKC (Okada 1996); PKC instead affects its sensitivity for calcium indirectly (Okada 1995), and exerts inhibitory rather than stimulatory effects (Riccio et al. 1996). Thus, NOS1 is not a likely candidate either. Finally, NOS3 is directly phosphorylated by PKC (Fleming et al. 2001; Chen et al. 2014) causing it to produce O_2_^−^ (Lin et al. 2003; Chen et al. 2014). Taking all this into account, our data suggest that NOS3 mediates NOS‐derived O_2_^−^ in response to acute Ang II stimulation. Studies with double knockouts that would provide a definitive answer to this question are far out of the scope of this paper.

In summary, we found that Ang II acutely causes O_2_^−^ production by NOS in thick ascending limbs. This process is dependent on PKC and NADPH oxidase and remains present in excess of L‐arginine. Stimulation of NO and O_2_^−^ by such factors as Ang II is important in regulating renal water and salt reabsorption. Understanding the processes involved in maintaining the balance between NO and O_2_^−^ may give insight into the pathogenesis and treatment of diseases associated with oxidative stress and Na retention.

## Conflict of Interest

None declared.
